# Cervical chondroid chordoma in a standard dachshund: a case report

**DOI:** 10.1186/1751-0147-53-55

**Published:** 2011-10-21

**Authors:** Øyvind Stigen, Nina Ottesen, Hans Gamlem, Caroline P Åkesson

**Affiliations:** 1 Department of Companion Animal Clinical Sciences, Norwegian School of Veterinary Science, PO Box 8146, NO-0033 Oslo, Norway; 2 Department of Animal Health, Section for Pathology, National Veterinary Institute, PO Box 750, NO-0106 Oslo, Norway; 3 Department of Basic Sciences, Norwegian School of Veterinary Science, PO Box 8146, NO-0033 Oslo, Norway

## Abstract

A ten-year-old male standard dachshund was presented with a history of neck pain and progressive gait disturbances. Following a neurological examination and diagnostic imaging, including CT, a neoplastic lesion involving the third and fourth cervical vertebrae was suspected. The lesion included an extradural mass on the right side of the spinal canal causing a local compression of the cervical cord. Surgery, using a modified dorsal laminectomy procedure, was performed in order to decompress the cervical spinal cord. Histopathological examination of the extradural mass indicated that the tumour was a chondroid chordoma. Following discharge, the quality of life for the dog was very good for a sustained period, but clinical signs recurred at 22 months. The dog was euthanased 25 months post-surgery. On post-mortem examination, a regrowth of neoplastic tissue was found to have infiltrated the bone and spinal cord at C3-C4. This is the first report to show that palliative surgery can offer successful long-lasting treatment of chondroid chordoma of the cervical spine in the dog.

## Background

Chordomas are slow-growing, locally destructive neoplasms arising in the cerebrospinal axis from remnants or derivatives of the notochord. These tumours are uncommon in humans and in animals, and only a few cases have been reported in dogs [1,2], cats [3,4], rats [5] and mink [6]. There are several reports of chordomas in ferrets where the tumour is typically located on the tail [7-9].

By clinicopathologic, immunohistochemical and DNA flow cytometric studies, three subtypes of chordomas are recognized in humans: 1) conventional chordoma, 2) chondroid chordoma and 3) chordoma with a malignant spindle cell component [10-12]. Distinct subtypes of chordomas have not been described in other species.

To the authors' knowledge, eight cases of chordomas or probable chordomas in dogs have been reported previously, and the locations of the tumours varied. Two were located in the cranial region [13,14], two within the cervical vertebral canal [15,16], two in the sacro-coccygeal area [13,17], one in the spinal cord at the level of L5-6 [18] and one was located between the trachea and ventral muscles of the first cervical vertebrae [19].

This is the first report describing surgical treatment and follow-up of a chordoma in the cervical spine of a dog.

## Case presentation

A ten-year-old, male, longhaired standard dachshund of 11 kg, presenting with lethargy and ataxia was referred to the Norwegian School of Veterinary Science for evaluation and treatment. It had a three month history of gradual behavioural change, reluctance to exercise and increasing stumbling on the right thoracic limb. A week before admission, the referring veterinarian also identified neck pain, a slight degree of general muscular atrophy and tetraparesis with an absence of the proprioceptive positioning response in all four limbs. The referring veterinarian then prescribed 10 mg prednisolone orally once daily for seven days.

### Clinical findings

On referral, the dog was bright and alert. Clinical examination revealed a reduced body condition including general muscular atrophy. The claws of the third and fourth digits were worn on all four limbs, especially on the right thoracic limb.

The dog was walked on a leash and ataxia and weakness of all four limbs were observed. The dog was able to walk up and down stairs and stumbling on a smooth floor was not observed. Signs of discomfort or spontaneous pain were not registered.

On neurological examination, cranial nerve function was normal except for a bilateral absence of the menace response that was explained by presence of keratitis and cataract in both eyes. The proprioceptive positioning response was absent in all four limbs. The hopping reaction and the tactile placing response were delayed in the right *versus *the left thoracic limb. On flexion of the neck, the dog showed signs of cervical pain.

The results of clinical biochemistry and a complete blood count were within the normal reference ranges for the laboratory.

### Diagnostic imaging

**Figure 1:**
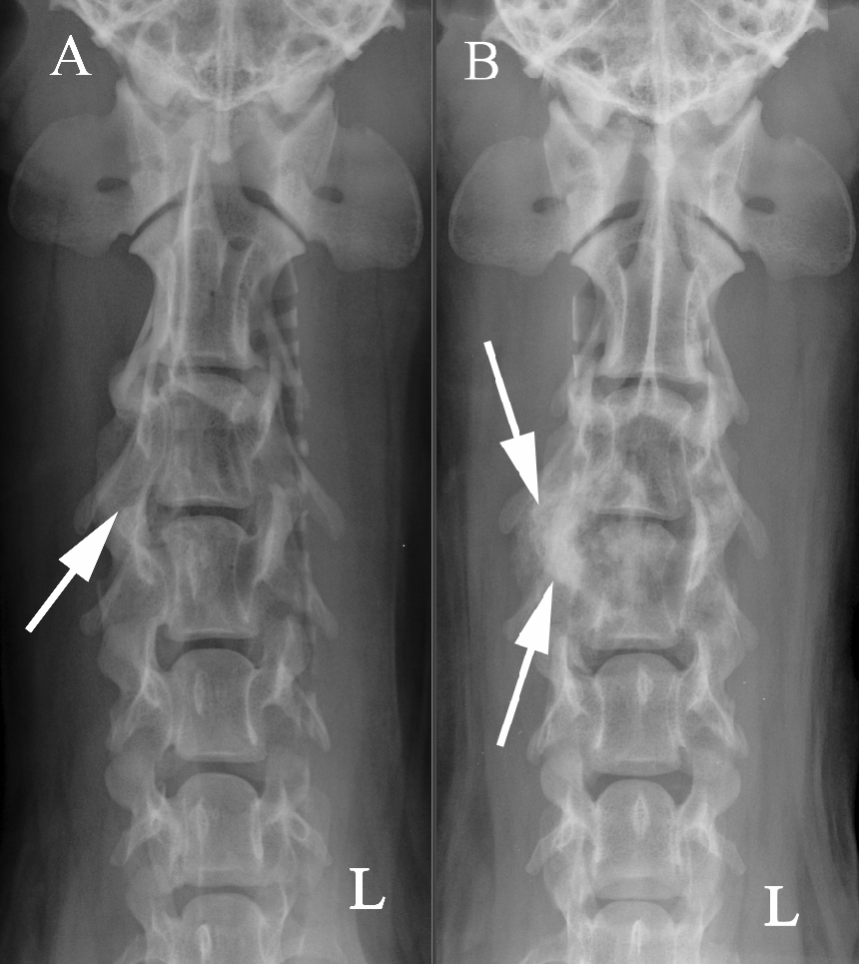
**Ventrodorsal radiographs of the cervical spine at the first presentation (A) and when the symptoms recurred 25 months later (B)**. There are mottled mineralized opacities at C3 and C4 and a mainly productive bone lesion (arrows) on the right side of the C3 vertebral arch.

The dog was premedicated with an im administration of 0·1 mg/kg acepromazine (Plegicil vet; Pharmaxim Sweden AB) and 0·2 mg/kg methadone hydrochloride (Metadon; NAF) and lateral and ventrodorsal radiographs of the cervical and thoracic vertebral column were taken. In the ventrodorsal view, a smoothly marginated expansive bone lesion was seen on the right side of the vertebral arch of C3 (Figure 1A). A moderate degree of calcification was identified in the intervertebral disc spaces C7-T1 and T8-9.

General anaesthesia was induced by an iv injection of 4 mg/kg propofol (Propofol-^® ^Lipuro B; Braun), maintained with 1·6 to 2 per cent isoflurane (Isoba vet; Schering Plough) in oxygen and air delivered by means of a circle patient breathing system. Computed tomography (CT) of the cervical spine was performed with the dog in ventral recumbency. The CT machine was a GE highspeed Fxi single slice helical scanner (GE Healthcare, Oslo Norway), the voltage and milliampere-seconds used were 120 kV and 150 mAs respectively and the slice thickness was 3 mm and 1 mm in both soft tissue (WW 400; WL 40) and bone (WW 1900; WL 580) algorithms.

**Figure 2:**
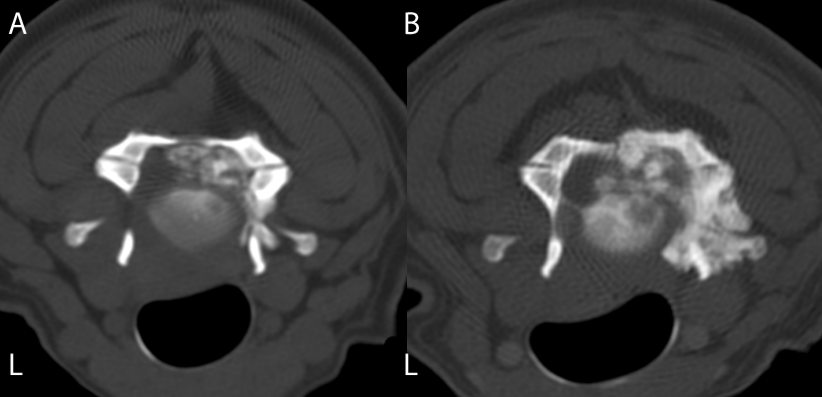
**CT scans in bone window through the C3-C4 intervertebral disc, before surgery (A) and 25 months post-surgery (B)**. (A) The image demonstrates calcified material centrally and to the right of the vertebral canal compressing the spinal cord to the left. (B) The image demonstrates local recurrence about two years after subtotal resection of the cervical chordoma.

**Figure 3:**
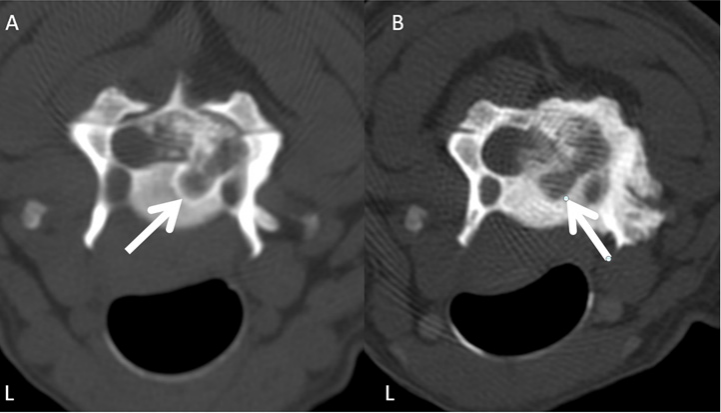
**CT scans in bone window through the cranial part of C4 at the first presentation (A) and 25 months later (B)**. (A) Note the radiolucent area with surrounding sclerosis (arrow) on the right dorsolateral side of the C4 body. (B) The C4 vertebral body has increased opacity and the radiolucent area is still visible (arrow).

CT revealed an extradural heterogenous and amorphous mass to the right of the vertebral canal just above the C3-C4 disc extending from mid C3 to mid C4 (Figure 2A and 3A). The mass caused lateral compression and displacement of two cervical cord segments to the left. Areas of high attenuation resembling calcification were scattered in the mass. In addition, there was calcified material and increased soft tissue density in the right intervertebral foramen around the C4 spinal nerve. A lytic lesion, surrounded by sclerosis, was observed on the right dorsolateral side of the cranial part of the C4 body. A corresponding lytic area was observed in the C3-C4 disc. Mild atrophy of the epaxial muscles (*m. splenius*, *m. semispinalis capitis *and *m. multifidus cervicis*) on the right side of the neck was also observed.

### Surgical treatment

Six days after the CT scan, surgery was performed. The dog was premedicated and anaesthesia inducted and maintained with the same procedure as presented in the Diagnostic Imaging section (above). For antimicrobial prophylaxis 40 mg/kg cephalothin (Cefalotin; ACS Dobfar Generics) was administered iv. Analgesic therapy included transdermal administration of 4·5 μg/kg/h fentanyl (Durogesic; Janssen Cilag).

The dog was placed in ventral recumbency with the head and neck gently flexed in a neutral position. A dorsal midline skin incision extending from the occipital protuberance to the dorsal spinous process of C6 was made. The laminae of vertebrae C2- C5 was exposed through an approach with lateral retraction of epaxial musculature to the level of the articular processes [20]. Periosteal bone formation was identified on the right parts of the C3 and C4 laminae. A dorsal laminectomy extending from the cranial third of C3 to the caudal third of C4 was performed using a high-speed spinal bur. The laminae were irregularly thickened and a mineralised mass was found extradurally to the right of a compressed, but otherwise normal looking dura mater and cervical cord. Epidural fat was almost absent. A sample of the mineralised mass and the adjacent laminae was collected for histopathology. Remaining abnormal tissue was removed to achieve satisfactory decompression, though some pathological tissue remained. A 2-3 mm thick layer of subcutaneous fat was placed at the laminectomy site before routine wound closure. A closed wound bandage was applied.

After surgery, the dog was monitored in the intensive care unit and given an iv injection of 4 mg/kg carprofen (Rimadyl; Pfizer, New York, USA). An im administration of 0·2 mg/kg methadone hydrochloride (Metadon; NAF) was repeated q4h in the immediate 12 hour-postoperative period. Anti-inflammatory treatment was continued by giving 2 mg/kg of carprofen po twice daily for two weeks. Two days post-surgery the dog was ambulatory with moderate ataxia and weakness in all four limbs. Spontaneous pain was not registered and urination was normal. The fentanyl patch was removed three days post-surgery.

The dog was discharged after six days. At that time, neurological function was similar to that observed preoperatively. Exercise restriction was advised, and the use of harness instead of a collar was recommended.

### Histopathological examination

The formalin fixed biopsy measuring 0·5 × 0·5 × 2 cm was decalcified and sectioned for histopathological examination. The morphological features together with immunohistochemical examinations indicated a chondroid chordoma.

### Postoperative development

The owner was questioned by telephone about the dog's condition twelve months after surgery. She reported that the dog was doing well without observable gait disturbances. The claws of the third and fourth digits of the right thoracic limb were normal. However, the owner commented that the dog tended to lie down when patted on his back and she presumed this behaviour was to avoid discomfort.

Twenty-five months following surgery, the owner telephoned and reported recurrence of clinical signs with gait disturbances and cervical pain. The dog had become progressively worse over the last three months, and despite treatment with carprofen, the owner felt the dog now was suffering. On readmission to the Norwegian School of Veterinary Science, the dog was depressed and reluctant to walk. Worn claws were seen on both thoracic limbs. When walked on a lead, the dog showed weakness (tetraparesis) and a mild lameness in the right thoracic limb. Hands-on examination of the head and neck revealed signs of pain, including attempts to bite the examiner. A systolic murmur was heard on both sides of the thorax.

The dog was sedated and a second radiographic study of the cervical vertebral column was performed. A mixed (destructive and productive) bone lesion was identified at C3 and C4, and included the body and arch of both vertebrae (Figure 1B). Areas of increased radiodensity were observed on the right side of the vertebrae, and the borders to soft tissue were without a marked periosteal response.

CT of the cervical spine was repeated with the dog in ventral recumbency. The slice thickness was 2 mm and 1 mm in both soft tissue and bone algorithms. The studies were performed before and after iv injection of 300 mg I/ml iohexol (Omnipaque; GE Healthcare, Oslo, Norway) at a dose of 1·8 ml/kg bodyweight.

The laminectomy of C3 and C4 was visible. The CT examination revealed a large mass to the right of the vertebral canal involving the C3 and C4 vertebral arches and the C4 vertebral body (Figure 2B and 3B). The mass was heterogeneous and it was not possible to distinguish fragments of possibly destroyed bone from tumour calcification. Two cervical cord segments (C4 and C5) were laterally compressed and displaced to the left.

Owing to the size, location and history of the cervical mass, the prognosis was considered to be poor and with the owner's consent the now twelve-year-old dog was euthanased and submitted for post-mortem examination.

### Pathological examination

**Figure 4:**
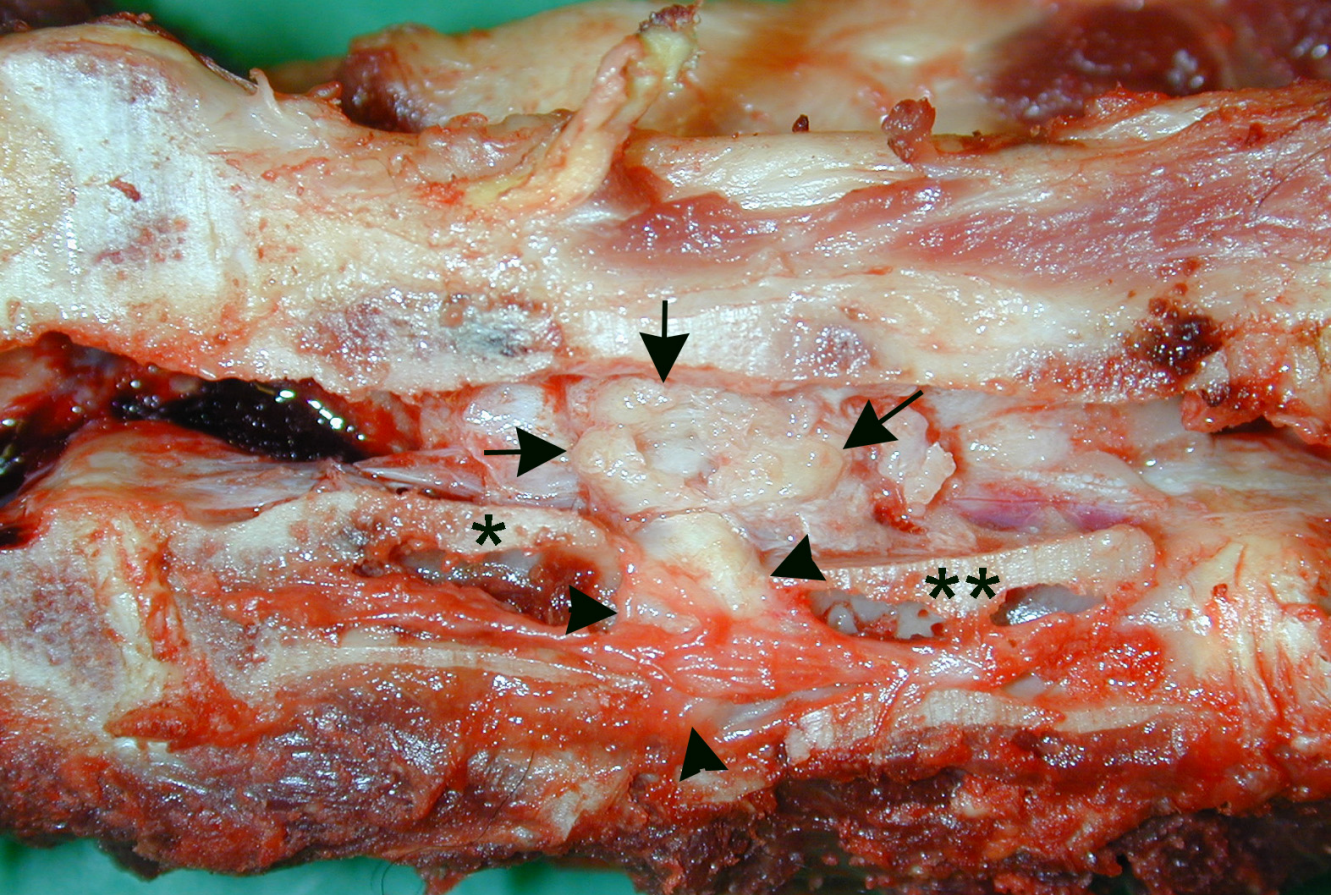
**Gross pathology of the cervical vertebral column**. The vertebral column is sectioned in the median plane. Tumour tissue (arrows) has replaced the intervertebral disc (arrowheads), joining the bodies of adjacent C3 (one asterisk) and C4 (two asterisks) vertebrae. The tumour extended laterally and dorsally on the right side of the cervical column and protruded into the vertebral canal where it adhered to the spinal cord (has been removed).

At post-mortem examination of the vertebral column, gross pathological findings were observed between cervical vertebrae C3 and C4 (Figure 4). The intervertebral disc was replaced by tumorous tissue which extended into, and diffusely infiltrated adjacent bone and soft tissues on the right lateral side of the vertebrae. The tumour measured approximately 3 × 3 × 4 cm, was relatively firm, multinodulated and with a glistening surface. On the right medial side of both C3 and C4 the tumour prominated into the vertebral canal and in an area of approximately 0·5 cm diameter it adhered to the spinal cord. The bodies of C3 and C4 were devoid of bone marrow, leaving a hollow cavity with only a string of soft tissue running through the length of the vertebral body. Metastases were not observed. In addition, the necropsy showed bilateral valvular endocardiosis and an intracranial tumour mass measuring 1 × 2 × 2 cm.

**Figure 5:**
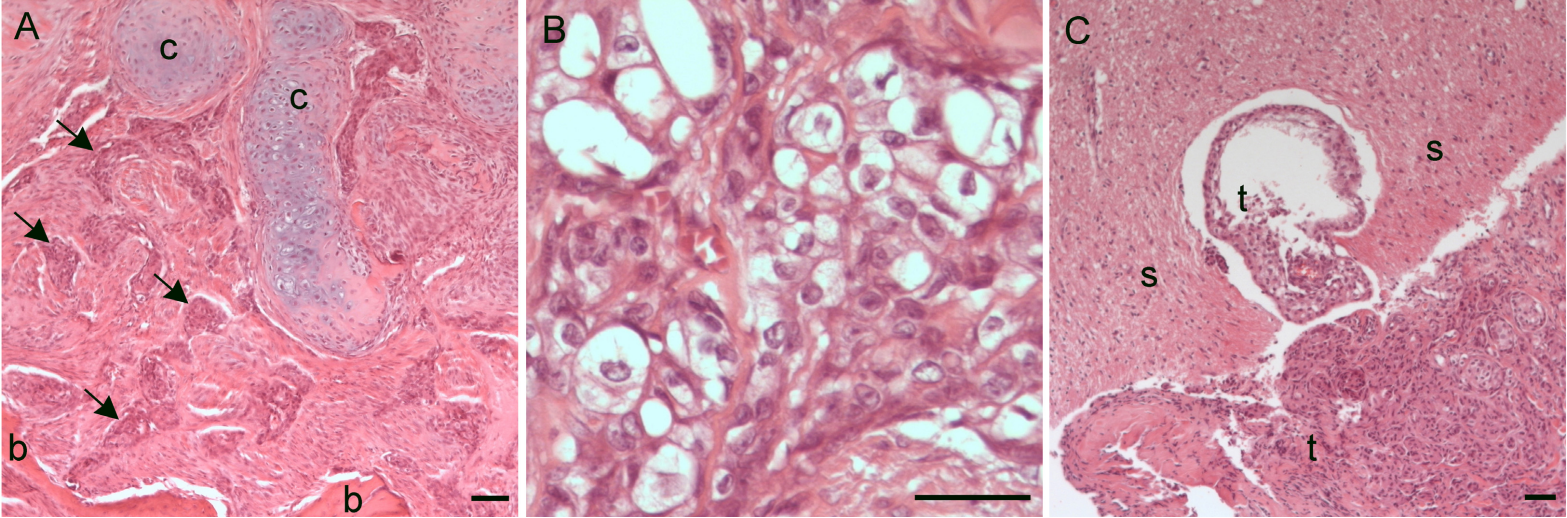
**Histology of the tumour in the cervical vertebral column**. (A) Section of tumour showing chordoid tumour cells in lacunaes (arrows) surrounded by connective tissue. The tumour infiltrated and replaced pre-existing, normally differentiated trabecular bone tissue (b). Multifocally there was chondroid differentiation in the tumour (c) (H&E, Bar 200 μm). (B) Physaliferous tumour cells, which are characteristic of chordomas, were observed multifocally (H&E, Bar 50 μm). (C) Section showing the tumour (t) to be locally invasive, here invading the spinal cord tissue (s) (H&E, Bar 200 μm).

Histopathological examination of the tissue from the area of C3 and C4 showed a poorly demarcated tumour consisting of neoplastic cells in lacunae separated by fibrous septa. The neoplastic cells were polygonal, moderately pleomorphic with poorly defined cell borders. The cells had moderate to abundant eosinophilic cytoplasm with small to medium sized nuclei that were round-, oval- or spindle shaped with a light to dark basophilic colour, and were interpreted as chordoid-like tumour cells (Figure 5A). In many areas of the tumour the cells contained cytoplasm that was markedly vacuolated, often with a centrally placed nucleus (physaliferous cells) (Figure 5B). A basophilic material was observed in a few areas and was interpreted as mucin matrix. There was chondroid differentiation multifocally (Figure 5A, c), which in some areas had a dark basophilic colour interpreted as mineralization. The trabecular bone tissue observed multifocally could consist of either pre-existing bone or newly formed bone tissue, indicating a remodelling process. The tumour showed irregular infiltrative growth into adjacent tissues, including preexisting trabecular bone tissue and the spinal cord (Figure 5C). The tumorous tissue also had a low mitotic index.

**Figure 6:**
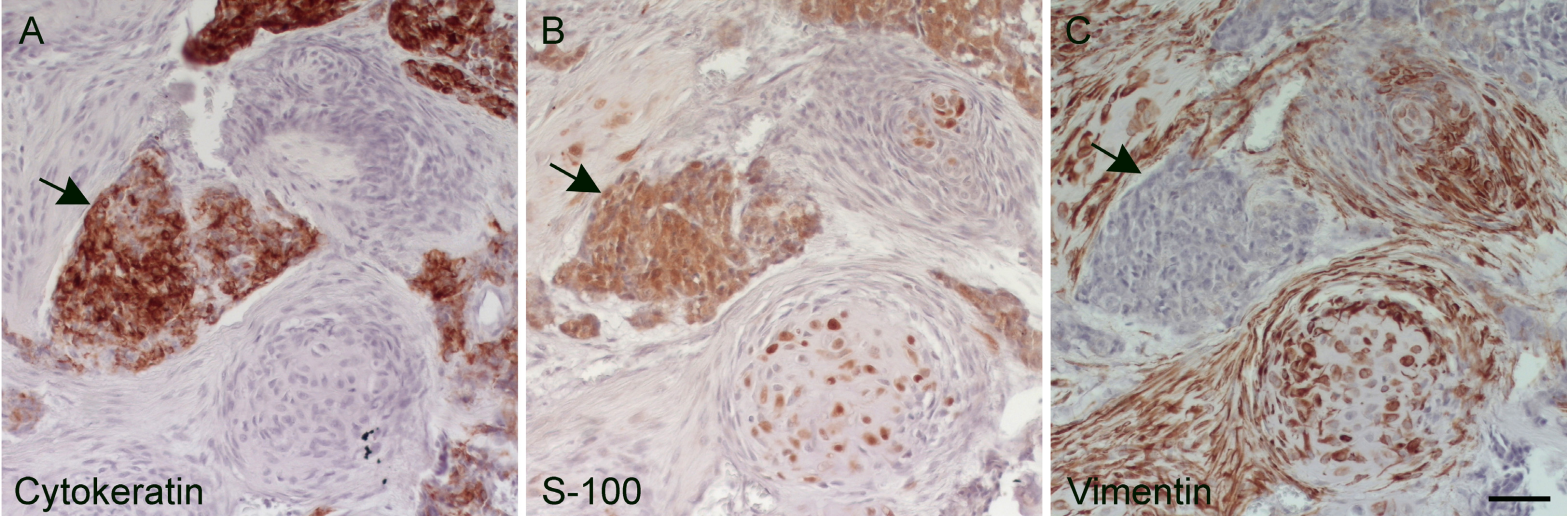
**Immunohistochemical staining of tumour tissue**. To differentiate this chondroid chordoma from chondrosarcoma, the tumour tissue was stained with vimentin, cytokeratin, and S-100. The chordoid tumour cells (arrows) showed a strong positive reaction for the cytokeratin- and S-100-antibody (A and B, respectively). The fibrous tissue and chondroid components were vimentin positive (C) (Bar 50 μm).

Immunohistochemical examination revealed strong labelling for cytokeratin and S-100 in the chordoid tumour cells (Figure 6A and 6B). Only a few of the cells showed weak labelling for vimentin (Figure 6C). The physaliferous cells contained PAS-positive material in the cytoplasm.

On the basis its location, the cellular components and the immunohistochemical results, the tumour was definitively diagnosed as a chondroid chordoma.

Histopathological and immunohistochemical examination of the intracranial tumour mass showed a meningioma with no resemblance to the tumour of the cervical spine.

## Discussion

Following surgery, which included decompression of the cervical spinal cord, ataxia and neck pain subsided and the symptoms did not recur for almost two years. The post-mortem examination revealed the chordoma to be only 3 × 3 × 4 cm in size and no metastases were observed. These findings are in accordance with previous reports that describe chordomas as slow-growing, locally aggressive neoplasms with a high rate of recurrence, but with a limited potensial for metastasis [7,21].

In this case, the aim of the surgery was to obtain a biopsy and to decompress the cervical spinal cord. Because it was not possible to remove all the pathologic (neoplastic) tissue, the decompression can be classified as palliative. However, following treatment the dog lived for two more years, including 22 months with a very good quality of life. This case shows that surgical treatment of a chondroid chordoma significantly increased the life span of a dog despite incomplete resection of the tumour. This finding indicates that surgical treatment of chondroid chordomas in the dog is warranted.

Surgical excision is considered to be the most important mode of treatment for chordomas in humans. Radiation therapy and chemotherapy are used as additional treatment modalities [22,23], but the delivery of sufficiently high doses of radiation to the tumour tissue is a challenge [24]. In future, molecular targeted chemotherapies may improve the treatment of chondromas.

In 55 cases of spheno-occipital chordomas in humans, Heffelfinger and others [21] found that the prognosis was better in 19 patients with chondroid chordomas *versus *in 36 patients with conventional chordomas. In dogs, distinct subtypes of chordomas have not been described and, to the authors' knowledge, no reports have included a follow-up after surgical treatment of canine chordomas. Therefore, the prognostic importance of the chondroid component in the chordoma presented herein is unknown.

In higher vertebrates, the nucleus pulposus is believed to be the only derivative of notochordal tissue [25]. However, remnants of notochord may persist outside the intervertebral discs anywhere along the vertebral column [26]. The chordoma described in the present case report included the C3-C4 disc space. The tumour could, therefore, have arisen from notochordal cells within the nucleus pulposus of the C3-4 disc. Previous reports concerning chordomas in dogs have not described pathology of intervertebral discs, indicating that notochordal tissue elements outside the discs as the origin of these tumours.

In chondrodystrophoid dogs, intervertebral disc degeneration is characterized by an early initiated and very accelerated metamorphosis of the nucleus pulposus from a mucoid to a chondroid type [27]. From the age of four years, the nuclei pulposi with very few exceptions are converted into the chondroid type, which implies the replacement of the notochordal cells. Hansen [27] studied the pathology of intervertebral discs in 206 chondrodystrophoid dogs, aged from under two months to over seven years. In the 75 dogs over four years of age, one seven year old dog was found to have mucoid nuclei pulposi in the cervical region. The presence of mucoid nuclei pulposi and thereby notochordal cells in some middle-aged chondrodystrophoid dogs combined with the slow-growing nature of chordomas can explain why this neoplasm can be diagnosed in a ten-year-old dachshund.

Besides physaliferous cells and the nest formation of chordoid tumour cells, in chordomas and not in cartilaginous tumours, immunohistochemical labelling is an important tool for differentiating these two tumour types and to confirm the diagnosis [28]. As was observed in the presented case, chordoma tumour cells label with the epithelial marker cytokeratin and S-100, a protein derived from the neural crest and chondrocytes among others. Chondrosarcomas stain positively for S-100 and vimentin a marker for mesenchymally derived tissues, but do not label with cytokeratin. Of the eight chordomas or probable chordomas previously reported in dogs, only three have been verified by immunohistochemical analysis [15-17].

## Conclusions

In dogs, chondroid chordomas may arise in the cervical spine and cause clinical signs from extradural masses that compress the spinal cord and spinal nerves. Both cervical vertebrae and discs may be subjected to neoplastic tissue. Sclerotic and lytic lesions could be identified by diagnostic imaging. Chondroid chordomas localized in the vertebral column may not be possible to remove by complete surgical excision. However, an incomplete resection including decompression of the spinal cord, may subsequently give a dog an excellent quality of life for almost two years.

## Consent

Written informed consent was obtained from the owner for publication of this case report and accompanying images. A copy of the written consent is available for review by the Editor-in-Chief of this journal.

## Competing interests

The authors declare that they have no competing interests.

## Authors' contributions

ØS carried out the clinical examination, performed the surgery and is the main author of the paper. NO carried out the diagnostic imaging. HG and CPÅ carried out the gross pathological, histopathological and immunohistochemical examinations. All authors read and approved the final manuscript.
